# Neurological and systemic effects of cocaine toxicity: A case report and review of the literature

**DOI:** 10.3892/mi.2024.196

**Published:** 2024-10-08

**Authors:** Zachary M. Hong, Julie A. Kromm

**Affiliations:** 1 Cumming School of Medicine, University of Calgary, Calgary, AB T2N 4N1, Canada; 2 Department of Critical Care Medicine, Cumming School of Medicine, Foothills Medical Centre, McCaig Tower, Calgary, AB T2N 5A1, Canada; 3 Department of Clinical Neurosciences, Cumming School of Medicine, Calgary, AB T2N 2T9, Canada; 4 Hotchkiss Brain Institute, University of Calgary, Calgary, AB T2N 4N1, Canada

**Keywords:** coma, cocaine, leukoencephalopathy, overdose, toxicity

## Abstract

While historically utilized as a topical anesthetic and active ingredient in a variety of tonics, beverages and cure-alls, cocaine is a commonly abused illicit substance. Cocaine use is associated with a variety of neurological and systemic complications. The present study describes the case of a 23-year-old female patient presenting with profound neurological and systemic complications of cocaine, including coma, leukoencephaloapthy, neurogenic stunned myocardium, transaminitis, acute kidney injury, rhabdomyolysis and mononeuropathy. The supportive management and subsequent outcomes of the patients are discussed in detail. The approach to coma is also discussed, including stabilization and supportive care, diagnostic considerations and management. Key considerations relating prognosticating disorders of consciousness are also highlighted.

## Introduction

Historically, cocaine was utilized as a topical anesthetic and active ingredient in a variety of tonics, beverages and cure-alls (1). At present, it is a commonly abused illicit substance. The United Nations estimates the global prevalence of cocaine use to be ~0.4% (2). Cocaine abuse results in several visits to emergency departments and hospitalizations, and is responsible for a variety of neurological and system complications. The present study reports the case of a patient presenting with systemic complications and significant neurological impairment secondary to cocaine use, who made a good recovery.

## Case report

A 23-year-old female presented with coma to a tertiary hospital in Canada. She last been seen in a normal condition 12-h prior and was subsequently found in bed. Collateral history revealed no preceding headache, psychiatric nor cognitive changes, focal neurological deficits, seizures, fever, neck discomfort, or any other systemic symptoms. There was no history of contact with any ill individuals, viral illness, travel, or vaccination. Her medical history disclosed no comorbidities and no prescribed medications. Her social history was significant for polysubstance use and subsequent information revealed she had recently attended a ‘pill party’.

Upon arrival to the emergency department, the patient had a temperature of 36.5˚C, a heart rate of 151 beats/min, a respiratory rate of 28 breaths/min, a blood pressure of 101/65, a Glasgow Coma Scale score of 4 and a glucose level of 6 mmol/l. Central painful stimuli produced extensor posturing, no verbal response and her eyes remained closed. The eyes were mid-position with no vertical eye movements on command, roving, bobbing/dipping, or gaze deviation. Her pupils were 2-3 mm and were reactive bilaterally with no hippus. A fundoscopy did not reveal any notable findings and there were no signs indicating papilledema. The remaining brainstem reflexes were intact. A more detailed motor examination revealed a normal tone throughout, 4+ reflexes symmetrically, bilateral clonus and an upgoing and equivocal plantar response on the right and left respectively. A detailed systemic examination revealed nuchal rigidity, normal otoscopy, no atypical odours and tachypnea with no irregular pattern; the respiratory examination yielded normal results; a cardiovascular examination also revealed normal results, including no murmurs; an abdominal examination did not reveal any notable findings and there were no extrahepatic signs of liver disease; there were also no concerning dermatological findings.

**Figure 1:**
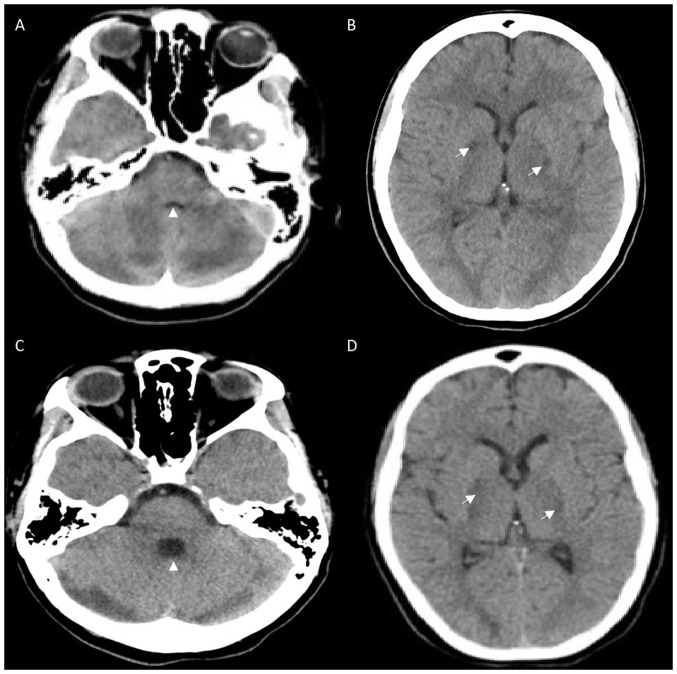
Computed tomography: (A and B) Day 1, and (C and D) day 13. (A) Loss of gray and white matter differentiation in the cerebellar hemispheres, with mass effect on the fourth ventricle (white arrowhead) are observed. (B) Bilateral hypodensities in the globus pallidus (white arrows) are observed. (C) The resolution of gray and white matter changes in the cerebellar hemispheres can be seen. No mass effect on fourth ventricle (white arrowhead) can be observed. (D) Decreased attenuation of bilateral hypodensities in the globus pallidus can be seen (white arrows).

Simultaneous with the assessment were resuscitative measures, including the administration of 2 mg naloxone with no effect; rapid sequence induction and endotracheal intubation; fluid resuscitation with normal saline; and the use of empiric acyclovir (10 mg/kg IV q8h), ceftriaxone (2 g IV q12h) and vancomycin (20 mg/kg IV load followed by 15 mg/kg IV q12h). Investigations (Table I) included a computed tomography (CT) scan of the head that revealed bilateral hypodensities in the globus pallidus, and the loss of gray-white differentiation in both cerebellar hemispheres (Fig. 1A and B). CT angiography revealed no venous or arterial abnormalities. While optic nerve sheath diameters on point of care ultrasound and CT head were <5 mm, the concern of mass effect within the posterior fossa precluded a lumbar puncture. Initial systemic investigations revealed acute kidney injury (AKI) with myoglobinuria and granular casts, transaminitis with preserved liver function, mild rhabdomyolysis and hypokinetic apex with mildly reduced left ventricle function (Table I).

The patient was admitted to the intensive care unit. For her systemic issues, fluid resuscitation continued, and the use of N-acetylcysteine was initiated with a bolus of 150 mg/kg followed by an infusion (50 mg/kg IV over 4 h followed by 100 mg/kg over 16 h). Ampicillin (2 g IV q4h) was added for possible rhombencephalitis. As dexamethasone was not administered prior to or with antibiotics, it was not initiated. High-dose thiamine (500 mg IV q8h) was initiated. Neuroprotective measures were maintained, including fever avoidance; mean arterial pressure augmentation with norepinephrine to 80 mmHg given the posterior fossa mass effect; the avoidance of hyponatremia; the maintenance of euglycemia; and the maintenance of the partial pressure of oxygen and carbon dioxide at 80-120 mmHg and 35-40 mmHg, respectively. An electroencephalography (EEG) revealed no seizures.

**Figure 2:**
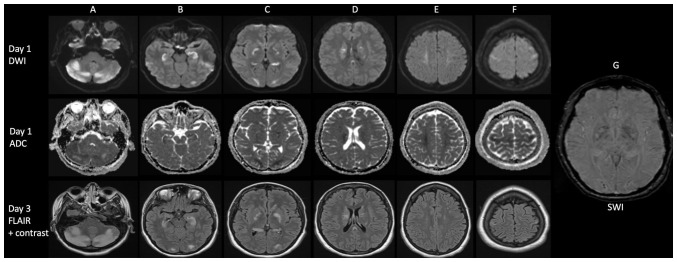
Magnetic resonance imaging of the patient on days 1 and 3. Day 1 magnetic resonance brain diffusion-weighted images, apparent diffusion coefficient sequences illustrating restricted diffusion involving the (A) bilateral cerebellar hemispheres, (B) hippocampi, and (C) globus pallidus and occipital lobes, (D) splenium of the corpus callosum and periventricular areas, (E) centrum semiovale, and (F) perirolandic regions. (G) Susceptibility weighted imaging sequences with petechial hemorrhage in occipital lobes and globus pallidus. (A-F, bottom row) Day 3 magnetic resonance brain fluid-attenuated inversion recovery (FLAIR) sequences illustrating the regression of restricted diffusion, increasing FLAIR hyperintensities and subtle enhancement in previously affected regions.

Once stabilized, efforts were directed at identifying an underlying etiology. An enhanced MRI of the brain at day 1 demonstrated restricted diffusion involving bilateral cerebellar hemispheres with posterior fossa mass effect and distended optic nerve sheaths, as well as restricted diffusion involving the bilateral hippocampus, globus pallidus, caudate nuclei, occipital lobes, periventricular region, the splenium of the corpus callosum, centrum semiovale and perirolandic area. Petechial hemorrhage was noted in the occipital lobes and globus pallidus (Fig. 2).

The urine toxicology was positive for cocaine, dextromethorphan, trazadone and zopiclone. Liquid chromatography-tandem mass spectrometry yielded positive results for: Benzoylecgonine, noroxycodone, morphine and hydromorphone. Tests for other substances, including levamisole, heroin, amphetamines, barbiturates, acetaminophen and salicylate yielded negative results. The ethanol level was negative and no osmolar or anion gaps were observed to suggest a toxic alcohol ingestion.

While a lumbar puncture was not able to be performed, given the high suspicion for a toxic-metabolic encephalopathy and unremarkable systemic work-up for concerning infectious etiologies (Table I), a central nervous system (CNS) infection was considered unlikely, and antivirals and antibiotics were discontinued with cautious monitoring. This also precluded cerebrospinal fluid analysis for possible inflammatory and autoimmune disorders, as well as systemic tests, the results of which were reassuring (Table I).

A follow-up enhanced MRI of the brain and cervical spine on day 3 revealed the complete regression of the restricted diffusion, although increasing FLAIR hyperintensities and subtle enhancement in the previously affected regions were observed with no new lesions within the brain or cervical spine (Fig. 2).

As a result of the aforementioned clinical assessment and investigations, cocaine toxicity was considered to explain both her neurological presentation and associated rhabdomyolysis, AKI, hepatitis and takotsubo cardiomyopathy.

With ongoing care, the systemic disturbances of the patient rapidly improved. Neurologically, the condition of the patient remained unaltered for 10 days until she began to spontaneously open her eyes and weakly withdraw. A follow-up CT scan of the head on day 13 revealed expected evolution (Fig. 1C and D). She began to localize with questionable tracking of visual stimuli on day 14 and obeyed simple motor commands shortly thereafter. Extensive discussions and shared decision-making with family members were performed regarding her diagnosis, uncertain prognosis and ongoing care. Due to ongoing somnolence, weakness and a resulting ineffective cough, a tracheostomy was required for safe liberation from mechanical ventilation to facilitate more time for possible recovery. At the time of transfer from the intensive care unit on day 16, she could obey simple commands, but had profound spasticity in all four extremities and left hemiparesis. More detailed assessments while recovering in the ward revealed severe cognitive impairment and aphasia.

She underwent intensive rehabilitation with occupational therapy and speech language pathology focused. Her spasticity was managed with baclofen and botox, and she underwent physical therapy to regain her strength. At 3 months following her presentation, a weakness of the left iliopsoas, quadricep and tibialis anterior muscles was observed, in addition to sensory disturbances involving the anteromedial thigh. Nerve conduction analyses and electromyography testing indicated a left lumbar plexus injury with extensive denervation. After a period of 3 months, she underwent surgery to reinnervate the left femoral neve using the obturator nerve, followed by progressive improvement in strength and mobility.

The patient returned home with support at 38 weeks following her presentation and continued with outpatient rehabilitation. At 1 year from her presentation, she was able to return to university part-time to work toward an engineering degree.

## Discussion

**Figure 3:**
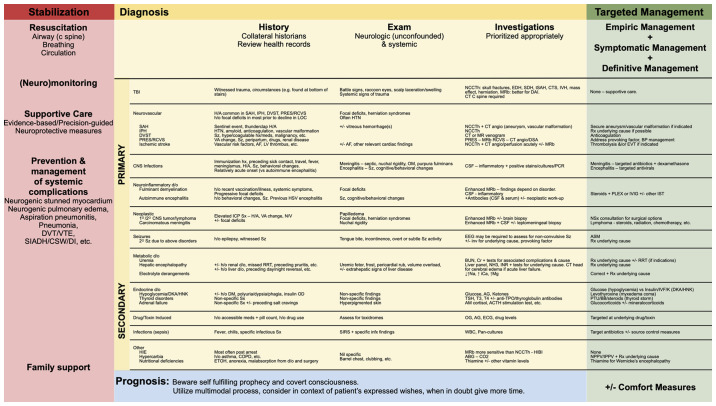
Clinical approach to the comatose patient: Stabilization, diagnosis (history, examination, investigations) and targeted management. Other considerations pertaining to the approach to an altered level of consciousness have been previously reported (3,4). ACTH, adrenocorticotropic hormone; AF, atrial fibrillation; AG, anion gap; ASM, anti-seizure medication; BB, beta blocker; BP, blood pressure; BUN, blood urea nitrogen; CNS, central nervous system; COPD, chronic obstructive pulmonary disease; CSF, cerebrospinal fluid; CT, computed tomography; CTS, contusion; DAI, diffuse axonal injury; DKA, diabetic ketoacidosis; d/o, diagnosis; DVST, dural venous sinus thrombosis; DVT, deep vein thrombosis; ECG, electrocardiography; EEG, electroencephalography; EDH, epidural hematoma; EtOH, ethanol; EV, endovascular thrombectomy; H/A, headache; HIBI, hypoxic ischemic brain injury; HIE, hepatic ischemic encephalopathy; HNK, hyperosmolar hyperglycemic non-ketotic coma; h/o, history of; HTN, hypertension; iCa, ionized calcium; infx, infectious; IPH, intraparenchymal hemorrhage; IPPV, intermittent positive-pressure ventilation; IST, immunosuppressive therapy; IVF, intravenous fluids; IVH, intraventricular hemorrhage; IVIG, intravenous immunoglobulin; LOC, level of consciousness; LV, left ventricle; MRb, MR brain; NCCTh, non-enhanced CT head; NPPV, non-invasive positive pressure ventilation; NSx, neurosurgical; N/V, nausea/vomiting; OG, osmolar gap; OM, otitis media; PLEX, plasma exchange; PNA, pneumonia; PRES, posterior reversible encephalopathic syndrome; PTU, propylthiouracil; RCVS, reversible cerebral vasoconstriction syndrome; RRT, renal placement therapy; Rx, treatment; SAH, subarachnoid hemorrhage; SDH, subdural hematoma; SIADH, syndrome of inappropriate antiduretic hormone secretion; SIRS, systemic inflammatory response syndrome; Sx, symptoms; Sz, seizure; tSAH, traumatic subarachnoid hemorrhage; T3, triiodothyronine; T4, thyroxine; TBI, traumatic brain injury; TPO, thyroid peroxidase; TSH, thyroid stimulating hormone; v/a, visual acuity; VTE, venous thromboembolism; WBC, white blood cells.

As regards the presentation of patients with coma (absent arousal and awareness), coma is the most severe syndrome on the disorders of consciousness spectrum and when presenting acutely must be regarded as a neurological emergency. Patients often lack overt signs of ongoing secondary injury and neurological decline that may only be detected with nuanced neurological exams or specialized non-invasive and invasive monitoring. This is in contrast to other medical emergencies and may give care providers a false sense of security. The approach to coma should be efficient, yet systematic and comprehensive. There are three parallel streams of thought and corresponding actions are required, including: i) Stabilization and supportive care; ii) diagnosis; and iii) management (Fig. 3).

Initial resuscitation should be focused on securing the airway (3). Cervical spine precautions should be maintained when trauma is a concern. When no anatomic airway difficulties are predicted, rapid sequence induction is preferred to avoid negative effects of the procedure on intracranial pressure. When time permits, a focused neurological examination should be performed prior, as it will be temporarily confounded by sedatives and paralytics. Hemodynamic stability must be ensured throughout intubation and thereafter to ensure adequate cerebral perfusion, while avoiding excessive hypertension. As demonstrated by the case described herein, patients may have concomitant cardiovascular issues potentially due to neurogenic stunned myocardium, a direct result of the causative disorder, or an unrelated comorbidity. Specific blood pressure parameters will depend on the clinical circumstances. Neuroprotective measures are a crucial and sometimes overlooked element of supportive care that are necessary to minimize secondary injury (4).

The differential diagnosis for causes of coma is broad (Fig. 3). These can be categorized into structural/primary neurological disorders and systemic/secondary neurological disorders and suggest that the former will often have focal signs, while the latter will not. There are exceptions to this rule, however. Patients with systemic/secondary neurological disorders may have focal deficits from prior diagnoses. Other systemic disorders have been reported to cause focal deficits (e.g., hypoglycemia). Given the relative limitations of the neurologic examination in coma, one may not detect subtle findings suggestive of structural disorders. This may also be confounded by medications utilized during resuscitation. As such, urgent neuroimaging should be obtained, unless there is an overt systemic cause.

Once neuroimaging excludes an acute vascular cause, empiric management for meningoencephalitis with acyclovir, antibiotics and dexamethasone may be prudent. Thiamine administration is also reasonable with a history of polysubstance abuse when little is known regarding the alcohol consumption of the patient. Antiseizure medications should only be administered in the case that seizures are confirmed either clinically or via EEG.

Following stabilization, extensive investigations and ongoing empiric and supportive care, the presumed cause for the coma in the patient in the present study was cocaine-induced CNS injury. Cocaine induced CNS injury occurs via three categories of pathophysiologic mechanisms. Vascular-mediated CNS damage occurs due to vasospasms, vasculitis, platelet aggregation and thrombus formation, cardioembolism and/or hypertension (5,6). Metabolic damage due to mitochondrial dysfunction causes demyelination, vacuolar degeneration, and axonal injury (5). Immune-mediated responses occur with or without levamisole. While levamisole can cause inflammation via the activation of dendritic cells, cocaine disrupts the endothelium, permitting migration of immune cells into the CNS and promotes increased levels of inflammatory cytokines (7).

Cocaine-induced CNS injury presents via a variety of mechanisms. With intoxication, patients can present with mydriasis, tics, fasciculations, vertigo, nausea and vomiting (8). Furthermore, the excessive stimulation of adrenergic and dopaminergic pathways can contribute to other neuropsychiatric sequelae, such as agitation, akathisia, formication and other hallucinations (8). Depending on the severity of symptoms, antipsychotic treatment may be indicated. Via vascular mechanisms, patients can experience isolated thunderclap headaches, reversible cerebral vasoconstriction syndrome, posterior reversible encephalopathy syndrome (PRES), and seizures and/or focal deficits in isolation (8,9). Cocaine-induced leukoencephalopathy resulting from metabolic and immune-mediated mechanisms often presents with cognitive impairments, and an altered level of consciousness including coma, impaired vision, and spasticity (5,10,11). The patient described herein presented predominantly with a leukoencephalopathy phenotype comprised of an altered level of consciousness, subsequent cognitive deficits and spasticity. While the initial coma could have been confounded by other medications (e.g., zopiclone, trazadone, narcotics) the lack of response to naloxone, neuroimaging, and delayed awakening suggests otherwise.

Imaging modalities are helpful in excluding other potential causes, assessing the degree of involvement, and for provide insight into the underlying pathophysiological mechanisms. Vascular mediated damage will result in infarctions, hemorrhage, arterial inflammation, occlusions and/or segmental vasoconstriction, in addition to patterns associated with PRES. Metabolic injury causes bihemispheric white matter (FLAIR and T2) hyperintensities, often with absent restricted diffusion, and absent gadolinium enhancement on MR brain (5). Subcortical U-fibers, the brainstem and cerebellum are often spared (5). MR spectroscopy demonstrates increased lactate and/or decreased N-acetylaspartate peaks (5). Immune-mediated pathology presents with subcortical and periventricular white matter (T2/FLAIR) hyperintensities, although with variable, diffusion-weighted signal abnormality, gadolinium enhancement and surrounding edema. However, case reports of brainstem, cerebellar and globus pallidus involvement have been noted (12,13). In the present study, the CNS injury of the patient appeared to be driven by all three pathophysiologic mechanisms, including immune mediated/inflammatory despite the absence of levamisole. This was evidenced by the following: The petechial hemorrhages in the occipital lobe; both vasogenic and cytotoxic edema; the involvement of regions highly susceptible to metabolic injury including the corpus callosum and globus pallidus; in addition to the subtle gadolinium enhancement.

In addition to the CNS effects, cocaine has been shown to be associated with a multitude of systemic complications (Table II) (4,14-17). The treating physician should consider the importance of managing the systemic issues of a patient, as further systemic decline will only predispose to worsening secondary neurological injury and outcomes.

Cocaine causes increased sympathetic tone and circulating catecholamines. This simultaneously increases oxygen demand via increased chronotropy, inotropy and afterload, while decreasing supply via coronary vasoconstriction, platelet adherence and thrombus formation. Local anesthetic effects and reduced sodium transport causes conduction abnormalities and reduced ventricular function (14). A number of these mechanisms may have been in effect in the patient described herein and/or neurogenic stunned myocardium. While the patient did demonstrate evidence of a type II myocardial infarction, therapies incorporated into proposed treatment algorithms (14), such as antiplatelets/anticoagulation and nitrates were not employed due to concerns regarding intracranial hemorrhage and posterior fossa mass effect and potential intracranial hypertension respectively.

The most common hepatic presentation is hepatic necrosis, accompanied by elevated serum aminotransferase and lactate dehydrogenase levels, as was managed in this patient. AKI most often results from rhabdomyolysis-induced acute tubular necrosis, but can also occur due to hypertension, vasoconstriction, thrombosis, infarctions and vasculitis (15). The AKI of the patient in the present study likely resulted from several of these mechanisms as her rhabdomyolysis was mild with no myoglobinuria and a downtime of 12-h without hyperthermia would not cause significant hypovolemia.

Rhabdomyolysis results from direct muscle toxicity, seizures and muscle ischemia due to arterial vasoconstriction, and compression in situations involving prolonged downtime. When severe, one must be vigilant to monitor for resulting complications, including compartment syndrome, hyperkalemia, hyperphosphatemia, hypocalcaemia in addition to AKI. Cocaine can also cause peripheral mononeuropathies, as became apparent in the course of the patient described herein. Mechanisms for this include arterial vasoconstriction, direct toxicity and/or compression (16,17).

There are also several direct and indirect drug-drug interactions with possible clinical implications in cocaine-users that physicians need to be aware of. These can occur due to changes in pharmacokinetics or pharmacodynamics, or due to genetic or epigenetic factors (18). The most important of which is the potential interaction with β-blockers. Cocaine promotes the release of norepinephrine and epinephrine. Τhe subsequent stimulation of β-1 receptors increases the heart rate and cardiac contractility, and β-2 receptors promote smooth muscle relaxation. α1 receptors, on the other hand, induce vasoconstriction. It has been suggested that in the context of stimulant use, β-blockers may lead to unopposed α-receptor stimulation, which can result in unopposed vasoconstriction, which in turn could cause coronary ischemia, hypertension and subsequent cardiovascular complications (18). However, this association has been recently called into question (19). Cocaine also induces an increase in serotonin synaptic activity, which may lead to the development of serotonin syndrome in the event that other serotonergic drugs are administered concurrently (e.g., fentanyl, linezolid, serotonin reuptake inhibitors). While cocaine is largely metabolized by serum esterases to inactive metabolites benzoylecgonine and ecgonine, a small portion undergoes hepatic N-demethylation by CYP3A4 to the active metabolite norcocaine, which is responsible for some of the toxic effects of cocaine. Several commonly prescribed medications are known inducers of CYP3A4 (e.g., phenytoin, carbamazepine) and may lead to increased levels of the toxic metabolite when used concurrently with cocaine (18). Additionally, the use of cocaine with acetylcholinesterase inhibitors, may lead to reduction of serum esterases and shunt cocaine metabolism toward the hepatic pathway, thus increasing norcocaine formation (18).

There is no consensus available to date on the treatment for cocaine-induced CNS injury. Treatment with steroids, intravenous immunoglobulin and plasmapheresis has been reported (5,11). The response to these therapies has been inconsistent. The patient in the present study received supportive care with early attention focused on preventing secondary brain injury and the management of complicating systemic factors. Later in the course, the focus shifted toward symptomatic management and rehabilitation.

Disorders of consciousness comprise a spectrum including delirium, minimally conscious state, unresponsive wakefulness syndrome and coma. While bedside clinical examinations are crucial, in patients with impaired consciousness, these are relatively basic, lack sensitivity and may have inter-examiner differences. Even careful standardized neurological assessments may misclassify conscious patients as unresponsive. Research regarding the detection of covert consciousness is emerging (20).

Impaired levels of consciousness may influence the decision to withdraw life-sustaining therapies in patients with acute brain injury, such as this when prognosis is uncertain. The potential to augment the accuracy of prognostication by various tests has been researched over the years and is perhaps best established within patients post-arrest. Often, however, evidence is limited in less common disorders (21) and research regarding covert consciousness that identify patients with better prognoses has yet to be translated into clinical practice (20). It is imperative that healthcare teams remain humble regarding their own knowledge; open regarding limitations in evidence; avoid personal biases that may cause inappropriate nihilism or optimism; and be vigilant of the self-fulfilling prophecy for the neuroprognostication of patients (22). Quality of life is subjective and multifactorial and shared decision-making with families is imperative. The patient in the present study subsequently obtained a good functional neurological outcome despite the profound acute presentation.

## Figures and Tables

**Table I:** Initial investigations performed for the patient in the present case report.

Category	Investigations	Results
Neurological	Computed tomography angiography- head and neck	Bilateral hypodensities in globus pallidus and loss of gray-white matter differentiation in cerebellar hemispheres. No venous or arterial abnormalities.
	Magnetic resonance imaging-brain	Edema involving cerebellum, globus pallidus, hippocampus, peri-rolandic, centrum semiovale, periventricular and occipital regions. Bilateral cerebellar infarcts and diffusion restriction in gray matter structures of cerebral hemispheres. Petechial hemorrhages in occipital lobes and splenium of corpus callosum.
	Electroencephalography	Diffuse alpha and delta wave slowing. No rhythmic or periodic patterns. No electrographic seizures.
Cardiac	Troponin	421 ng/l
	Electrocardiogram	Sinus tachycardia with no other abnormalities
	Transthoracic echocardiogram	Hypokinetic apex with low-normal left ventricular systolic function (53.6%)
Respiratory	Venous blood gas	pH 7.33
		PaCO_2_ 47 mmHg
Hepatic	Alanine transaminase	1562 U/l
	Aspartate aminotransferase	1037 U/l
	Gamma-glutamyltransferase	105 U/l
	Alkaline phosphatase	Normal
	Bilirubin-total and direct	Normal
	Abdominal Ultrasound	Mild hepatomegaly and splenomegaly, mild increase in liver echogenicity, edematous gallbladder. Normal hepatic and portal vein blood flow via doppler
	Ammonia	Normal
Pancreatic	Lipase	173 U/l
Renal	Creatinine	Normal
	Blood urea nitrogen	Normal
	Urinalysis	Positive for myoglobinuria and granular casts
Metabolic	Sodium	Normal
	Potassium	
	Phosphorus	
	Magnesium	
	Calcium	
	Lactate dehydrogenase	2,500 U/l
	Glucose	Normal
	Thyroid stimulating hormone	Normal
	Cortisol	Normal
	Vitamin B12	Normal
Musculoskeletal	Creatinine kinase	1,162 U/l
Hematological	Hemoglobin; white blood cells; platelets	Normal
	International normalized ratio; partial thromboplastin time	Normal
	Lupus anticoagulant testing; anti-cardiolipin antibodies; anti-β-2 glycoprotein antibodies	Negative
Toxicological	Acetaminophen and salicylate levels	Negative
	Ethanol level	Negative
	Anion gap/osmolar gap	Negative-therefore no toxic alcohol testing sent
	Toxicological panel	Positive: Cocaine, benzoylecgonine (782 ng/l), hydromorphone (1,166 ng/l), morphine (144 ng/l), noroxycodone (61 ng/l), dextromethorphan, zopiclone, trazadone, lidocaine.
		Negative: Amphetamine, 3,4-methylenedioxyamphetamine, methadone, 2-ethylidine-1,5-dimethyl-3,3-diphenylpyrrolidine, fentanyl, norfentanyl, oxycodone, codeine, norcodeine, buprenorphine, norbuprenorphine, hydrocodone, 6-monoacetylmorphine
Infectious	Blood cultures	Negative
	Sputum culture	Negative
	Viral swabs	Negative for COVID-19, influenza A/B, respiratory syncytial virus A/B, adenovirus, metapneumovirus, parainfluenza, enterovirus/rhinovirus, coronavirus
	Mycoplasma Pneumonia PCR	Negative
	Urine culture	Negative
	Legionella antigen	Negative
	Hepatitis A, B, C, E serology	Negative
	Human immunodeficiency virus	Negative
	Anti-Epstein-Barr virus viral capsid antigen IgM/IgG; Anti-Epstein-Barr virus nuclear antigen 1 IgG	Negative
	Cytomegalovirus IgM	Negative
Inflammatory and demyelinating	C-reactive protein	Normal
	Anti-nuclear antibodies	Negative
	Anti-neutrophilic cytoplasmic anti-bodies	Negative
	Extractable nuclear antigen anti-bodies	Negative
	Immunoglobulin levels-M, G, A	Normal
	Complement levels-C3, C4	Normal
	Anti-	Negative
	N-methyl-D aspartate (NMDA) Voltage gated potassium channel (VGKC)	
	Glutamic acid decarboxylase 65 (GAD65)	
	GABA-aminobutyric acid (GABA) Myelin oligodendrocyte glyco-protein	
	(MOG)	
	Aquaporin 4 (AQP4)	
	Glomerular basement membrane antibody (GBM)	
	Myelin-associated glycoprotein neuropathy (MAG)	

**Table II:** Complications of cocaine toxicity.

System	Mechanism	Clinical manifestations	(Refs.)
CNS	Vascular Mediated: Vasospasm, vasculitis, platelet aggregation, thrombus formation, cardio-embolism, hypertension Metabolic Damage: Mitochondrial dysfunction, vacuolar degeneration, demyelination, axonal injury Immune-Mediated: Activation of dendritic cells, disruption of endothelium permitting immune cell migration into CNS, increased levels of inflammatory cytokines	Acute	(5,8-11)
		Reversible cerebral vasoconstrictive syndrome (RCVS)	
		Posterior reversible encephalopathy syndrome (PRES)	
		Hemorrhagic and ischemic strokes	
		Leukoencephalopathy	
		Resulting in:	
		Altered level of consciousness	
		Seizures	
		Focal neurological deficits	
		Spasticity	
		Headache	
		Chronic	
		Movement Disorders-Tourette's syndrome, dystonia, tardive dyskinesia, chorea, akathisia	
		Cognitive deficits	
		Spasticity, focal neurological deficits	
Cardiovascular	↑ Sympathetic tone/circulating catecholamines	Myocardial infarction	(14)
	↑ Oxygen demand via ↑ inotropy, chronotropy and afterload	Aortic dissection	
	Coronary vasoconstriction, platelet adherence and thrombus formation	Infective endocarditis	
	Conduction abnormalities (↑PR, QRS, QTc intervals)	Reduced systolic and diastolic dysfunction	
		Arrhythmias	
Respiratory	Pulmonary vasoconstriction	Pulmonary hypertension and right heart failure	(15)
	Other vascular mediated effects including vasculitis, platelet aggregation, thrombus formation, etc.	Acute respiratory distress syndrome (ARDS)	
		Diffuse alveolar hemorrhage	
		Pneumothorax, pneumomediastinum	
		Organizing pneumonias	
	Bronchoconstriction	Pulmonary edema	
	Immune-mediated effects	Pneumonia, lung abscess, empyema	
	Introduction of infections		
Gastrointestinal	Mesenteric ischemia due to vasospasm, vasculitis, platelet aggregation, thrombus formation, and/or cardio-embolism	Ischemic bowel	(15)
		Intestinal perforations	
		Gastric ulcerations	
		Retroperitoneal fibrosis	
Hepatic	Hepatic ischemia and/or necrosis from vasospasm, vasculitis, platelet aggregation, and thrombus formation	Transaminitis, varying degrees of liver failure	(15)
Renal	Rhabdomyolysis, hypertension, vasoconstriction, thrombosis, infarctions, and vasculitis	Acute kidney injury, renal failure	(15)
PNS	Direct muscle toxicity, seizures and muscle ischemia from arterial vasoconstriction and compression from prolonged downtime Arterial vasoconstriction, direct toxicity and/or compression	Rhabdomyolysis	(9,16,17)
		Peripheral neuropathies	
Dermatological	Vasospasm, vasculitis, platelet aggregation, thrombus formation and immune activation	Blackened hyperkeratotic palms (‘crack hands’)	(6)
		Acute multifocal skin necrosis	
		Acute generalized exanthematous pustulosis	
		Cutaneous fibrosis	
		Chronic skin ulcers	
		Scleroderma	
		Cocaine-related bullous disease	
		Buerger disease	
		Pseudovasculitis	
		Urticarial vasculitis	
		Eosinophilic granulomatosis polyangitis	
		IgA vasculitis	
		Necrotizing granulomatous vasculitis and necrotizing vasculitis	
		Steven-Johnson syndrome	
Head and neck	Nasal and palatal ischemia and necrosis	Nasal septum perforation	(15)
		Nasal bone osteomyelitis	
		Nasal solid tumors or lymphoma	
		Dental carries	
		Palatal necrosis and/or perforation	
Psychiatric	Facilitation of dopamine neurotra-nsmission involving D-1, D-2 and D-3 receptors	Euphoria, psychosis, agitation, panic, paranoia	(15)
		Crash phase lasting several hours-anxiety, depression, drug craving, exhaustion, hypersomnolence	
Other	Increased motor activity, increased heat production, reduced heat dissipation (due to vasoconstriction)	Hyperthermia	(15)

## Data Availability

The datasets used and/or analyzed during the current study are available from the corresponding author on reasonable request.
